# GREB1L overexpression is associated with good clinical outcomes in breast cancer

**DOI:** 10.1186/s40001-023-01483-y

**Published:** 2023-11-14

**Authors:** Ke Dong, Chenchen Geng, Xiaohong Zhan, Zhi Sun, Qian Pu, Peng Li, Haiyun Song, Guanghui Zhao, Haidong Gao

**Affiliations:** 1 Department of Breast Surgery, Qilu Hospital of Shandong University (Qingdao), No. 758 Hefei Road, Qingdao, 266000 Shandong China; 2 Department of Ultrasound, Qilu Hospital of Shandong University (Qingdao), No. 758 Hefei Road, Qingdao, 266000 Shandong China; 3 Department of Pathology, The Affiliated Hospital of Qingdao University, No. 58 Haier Road, Qingdao, 266000 Shandong China; 4 Department of Breast Diseases, Shandong Second Provincial General Hospital, No. 4 Duanxing West Road, Jinan, 250000 Shandong China; 5 Department of Pathology, Qilu Hospital of Shandong University (Qingdao), No. 758 Hefei Road, Qingdao, 266000 Shandong China; 6 Women and Children’s Hospital, Peking University People’s Hospital (Qingdao), No. 7, Jinsheng 1St Road, Qingdao, 266111 Shandong China; 7 Medical Laboratory Center, Qilu Hospital of Shandong University (Qingdao), No. 758 Hefei Road, Qingdao, 266000 Shandong China; 8 Oncology Laboratory, Qilu Hospital of Shandong University (Qingdao), No. 758 Hefei Road, Qingdao, 266000 Shandong China

**Keywords:** Breast cancer, GREB1L, Hedgehog, Prognosis

## Abstract

**Background:**

Breast cancer is the most common malignant tumor among women worldwide. GREB1L is a protein-coding gene. Previous studies have shown that GREB1L plays a vital role in lung and gastric adenocarcinoma. Currently, there is no relevant report about its role in breast cancer.

**Methods:**

The Cancer Genome Atlas database was used to compare the expression level of GREB1L between tumor and normal tissues. The TISIDB website was used for prognosis analysis. The LinkedOmics database was used to predict the potential biological mechanism of GREB1L in breast cancer. Immunohistochemistry was used to detect the GREB1L expression level in breast tissue. Western blotting was used to detect the GREB1L expression level in cell lines. Transwell assays, CCK-8 cell proliferation assays, and colony formation assays were used to detect the migration, invasion, proliferation, and colony formation abilities of cells. Subcutaneous xenograft models were used to detect the in vivo tumor formation abilities of cells.

**Results:**

GREB1L is highly expressed in breast cancer tissues and breast cancer cells. KEGG enrichment analysis suggested that GREB1L participates in the regulation of the Hedgehog signaling pathway; changes in GREB1L expression affected the migration and invasion abilities of MCF7 and MDA-MB-231 cells. Although changes in GREB1L expression did not affect their proliferation and colony formation abilities in vitro and in vivo, they affected the expression of tumor metastasis-related genes in vivo. The overexpression of GREB1L in breast cancer predicted a favorable prognosis.

**Conclusion:**

These results showed that GREB1L is involved in the development of breast cancer, and it may be a potential molecular marker for predicting the prognosis of breast cancer.

**Supplementary Information:**

The online version contains supplementary material available at 10.1186/s40001-023-01483-y.

## Introduction

Currently, breast cancer (BRCA) is the most common malignant tumor among women [1]. The main treatment for BRCA is surgery, supplemented with radiotherapy, chemotherapy, and endocrine therapy. In addition, targeted molecular drugs are also emerging; these include drugs targeting human epidermal growth factor receptor-2 (HER2), inhibitors of poly ADP ribose polymerase (PARP), and inhibitors of the phosphoinositide 3-kinase/AKT/mammalian target of rapamycin (PI3K/Akt/mTOR) pathway [2–5]. The emergence of these drugs has significantly prolonged the survival of patients. However, as BRCA is phenotypically and functionally heterogeneous, further research is still needed to identify new prognostic markers and therapeutic targets [6].

Growth regulation by estrogen in breast cancer 1 like (GREB1L), an estrogen-regulated gene, is a coactivator of the retinoic acid receptor (RAR) gene, the activation of which regulates the RAR pathway. RAR signaling has been confirmed to trigger Müllerian epithelial cell differentiation and establish the border between the uterus and vagina. Thus, GREB1L plays an important role in developing the embryonic metanephros and reproductive tract [7]. Additionally, some studies have shown that the mutation of GREB1L also plays a vital role in nonsyndromic inner ear malformations and deafness, familial and sporadic hereditary urogenital a dysplasia, and Mayer–Rokitansky–Kuster–Hauser syndrome [8, 9]. Recent bioinformatics research suggested that GREB1L was associated with immune regulation and methylation in gastric and lung adenocarcinoma. Moreover, GREB1L is a novel predictive and prognostic biomarker of gastric and lung adenocarcinoma [10, 11]. These studies suggest that GREB1L has multifaceted underlying functions and mechanisms in different malignancies. However, the relationship between GREB1L and BRCA is still unclear.

In our study, we used bioinformatics approaches, such as differential expression analysis, Kaplan‒Meier survival analysis, multivariate Cox regression analysis, coexpression gene analysis, and gene set enrichment analysis (GSEA), to reveal that GREB1L is highly expressed in BRCA tissues compared with normal breast tissues and that its upregulation is associated with a good prognosis. Moreover, we verified that the expression of GREB1L in BRCA tissues was higher than that in paired adjacent tissues, and its expression in BRCA cells was higher than that in normal mammary epithelial cells. Transwell assays proved that knockdown of GREB1L expression promoted cell migration and invasion. Subcutaneous xenograft models showed that GREB1L can affect the expression of tumor metastasis-related genes. These findings suggest that GREB1L plays an important role in predicting the prognosis of BRCA and preliminarily clarified its function in BRCA.

## Material and methods

### Data source and online analysis tool

Gene expression data with clinical information (Workflow Type: HTSeq-TPM and HTSeq-FPKM) were acquired from The Cancer Genome Atlas (TCGA) database (https://portal.gdc.cancer.gov/) [12]. Samples without sufficient clinical information were excluded. A total of 8626 pancancer tissues and 713 corresponding paracancerous normal tissues from the TCGA-ALL dataset and 1083 tumor tissues and 111 paracancerous normal tissues from the TCGA-BRCA dataset, including 110 paired samples, were enrolled in this study. R (3.6.3 version) with the ggplot2 package (3.3.3 version), survminer package (0.4.9 version), survival package (3.2–10 version), pROC package (1.17.0.1 version), RMS package (6.2–10 version), and GSVA package (1.34.0 version) were used to process the original data and generate some figures and tables. Three online databases, including LinkedOmics (http://www.linkedomics.org/login.php), TISIDB (http://cis.hku.hk/TISIDB/index.php), and Kaplan‒Meier Plotter (http://kmplot.com/analysis/), were applied in this study.

### Differential expression analysis

We proceeded with logistic regression analysis of the correlation between GREB1L mRNA expression and clinical characteristics in BRCA. We compared GREB1L expression levels between tumors and corresponding paracancerous normal tissues in 23 human tumor types via the Wilcoxon rank sum test. In the BRCA cohort, we analyzed GREB1L expression levels between tumor tissues and paracancerous normal tissues. The expression levels in paired samples were also explored. The results are shown in scatter plots.

### Prognostic value

According to the median value of GREB1L expression, patients were classified into low- and high-expression groups. The TISIDB website was used to create a bar plot to compare the association between GREB1L expression and overall survival (OS) across 30 human malignancies. Data were processed by the log-rank test. Kaplan‒Meier curves of OS and relapse-free survival (RFS) between the GREB1L low- and high-expression groups were generated based on gene chip data from the Kaplan‒Meier plotter database. The adopted statistical approach was also the log-rank test. A forest plot of the multivariate Cox regression analysis was processed to show the prognostic factors in BRCA. We calculated the *p* value, hazard ratio (HR), and 95% confidence interval (CI) of every potential predictor. Prognostic factors with HR > 1 and *p* < 0.05 were risk factors for BRCA prognosis. Moreover, those with HR < 1 and *p* < 0.05 were regarded as protective factors.

### Coexpression networks and gene set enrichment analysis

To predict the potential biological mechanism of GREB1L in BRCA, we used the LinkFinder module in the LinkedOmics website (http://www.linkedomics.org/login.php) to research the coexpression network of GREB1L in the TCGA-BRCA dataset. Then, in the LinkInterpreter module of the same portal, GSEA was utilized to identify terms significantly related (FDR < 0.05) to GREB1L coexpressed genes. The analysis contains four aspects, including Gene Ontology biological process (GO-BP), Gene Ontology cellular component (GO-CC), Gene Ontology molecular function (GO-MF), and Kyoto Encyclopedia of Genes and Genomes (KEGG) pathway analysis. We utilized Pearson correlation as a statistical approach, and a false discovery rate (FDR) < 0.05 was considered to indicate significant correlations or enrichment.

### Specimens and cells

Breast tissue specimens were obtained from Qilu Hospital of Shandong University (Qingdao). The specimens were frozen in liquid nitrogen immediately after surgical resection. All breast tissues were collected according to the protocol approved by the Ethics Committee of Shandong University Qilu Hospital (Qingdao). MCF10A, MCF7, Hs578T, ZR-75-1, MDA-MB-231, MDA-MB-453, and SK-BR-3 cells were preserved by our laboratory. Hs578T cells were cultured in Dulbecco’s modified Eagle’s medium (DMEM) (BI, C3113) containing 10 μg g/ml insulin. MCF10A cells were cultured in DMEM/F12 containing 5% horse serum, 20 ng/ml epidermal growth factor, 0.5 μg/ml hydrocortisone, 10 μg/ml insulin, and 1% nonessential amino acids (Procell, CM-0525). ZR-751 cells were cultured in Roswell Park Memorial Institute (RPMI)-1640 (Procell, PM150110) medium. MDA-MB-453 cells were cultured in Leibovitz’s L-15 (Procell, PM151010) medium. MDA-MB-231, MCF7, and SK-BR-3 cells were cultured in DMEM. All cell culture media included 10% fetal bovine serum (FBS) (BI, C04001) and 1% penicillin‒streptomycin solution (Procell, PB180120).

### Lentiviral construction and transfection

Lentiviruses carrying short hairpin RNA (shRNA) against GREB1L were constructed by Genechem Company (Shanghai, China). The RNAi sequence targeting human GREB1L was 5′- GCGTTTGGTATCACTGTGTAT-3′. The negative control sequence was 5′-TTCTCCGAACGTGTCACGT-3′. Viruses were transfected with HitransG P according to the manufacturer’s instructions.

### Transwell migration and invasion assays

Transwell migration and invasion assays were performed using 24-well insert transwell chambers (Corning, #3422). Approximately 5 × 10^4^ MCF7 cells and 3.5 × 10^4^ MDA-MB-231 cells were resuspended in 200 µl DMEM without FBS and seeded in the upper chamber. DMEM containing 20% FBS was added to the bottom wells to stimulate migration or invasion. For the migration assay, the seeded MCF7 cells were incubated for 24 h (MDA-MB-231 cells for 12 h). For the invasion assay, MCF7 cells were incubated for 24 h (MDA-MB-231 cells for 18 h). After incubation, the cells on the upper surface of the chamber were wiped off with a cotton swab and then rinsed with PBS, and the cells on the lower surface of the chamber were fixed with methanol and stained with 0.1% crystal violet. Then, the cells were counted at 100 × with a microscope. For the invasion assay, the top chamber was coated with Matrigel (ABW, 082704).

### Cell counting kit-8 (CCK-8) cell proliferation assays

Approximately 3 × 10^3^ MDA-MB-231 cells per well or 4 × 10^3^ MCF7 cells per well were seeded in 96-well plates, and 10 µl CCK-8 (Dojindo, CK04) reagent was added to each well before measurement. After incubation for 2 h in a 37 ℃ incubator, the absorbance at 450 nm (OD450) was measured. The cells were assessed once every 24 h.

### Colony formation assays

Approximately 1 × 10^3^ cells were seeded in six-well plates. After 2–3 weeks of treatment with puromycin, the cells were washed three times with PBS, fixed with methanol and stained with 0.1% crystal violet.

#### Subcutaneous xenograft models in vivo

Approximately 3 × 10^6^ MDA-MB-231 cells were subcutaneously injected into the flanks of 6-week-old female BALB/c nude mice (*n* = 6 per group, Charles River, Beijing, China). The tumor diameter in the nude mice was measured every 5 days. The mice were sacrificed at 6 weeks, and the tumor weights and volumes were measured.

#### Real‑time quantitative PCR

Total RNAs were extracted from cells using TRIzol reagent (TIANGEN Biotech, Beijing, China) and reverse-transcribed into cDNA using All-In-One 5X RT MasterMix (abm, Vancouver, Canada). Then, qPCR was performed using BlasTaqTM 2X qPCR MasterMix (Abm, Vancouver, Canada). The primers were synthesized by Sangon Biotech (Shanghai, China). The following gene-specific primers were used: GAPDH, forward 5′-GGAGCGAGATCCCTCCAAAAT-3′, reverse 5′-GGCTGTTGTCATACTTCTCATGG-3′; GREB1L, forward 5′-GCTCTAGCAATGAGGTTCACTGG-3′, reverse 5′-GTCTCGTCACATCTCAGAAGTGG-3′.

### Western blotting

RIPA lysis buffer (Solarbio, #R0010) was used to extract total cell protein. PMSF (Solarbio, P0100) was added to the lysis buffer. Lysates were separated into 6% and 10% acrylamide gels. Then, the proteins were transferred from the gel to a PVDF membrane (Immobilon-P, IPVH00010). A blocking buffer (Boster, AR0041) was used to block the blots. Anti-GREB1L (1:300, ATLAS ANTIBODIES, HPA041647), anti-β-Tubulin (1:1000, absin, abs830032), E-cadherin (1:1000, Cell Signaling Technology, 14472S), N-cadherin (1:1000, Cell Signaling Technology, 13116S), and Vimentin (1:1000, Cell Signaling Technology, 5471S) were used as the primary antibodies. Anti-β-Tubulin was used as an internal control. HRP-goat anti-mouse IgG (1:5000, Earthox, E030110) and HRP-goat anti-rabbit IgG (1:5000, Earthox, E030120) were used as the secondary antibodies.

### Immunohistochemistry

Immunohistochemistry (IHC) was carried out as described previously [13] with anti-GREB1L (1:300, ATLAS ANTIBODIES, HPA041647) in paraffin-embedded breast cancer tissue sections.

### Statistical analysis

All experiments were repeated three or more times. All quantitative data are presented as the means ± SDs. We used a standard two-tailed unpaired t test for the statistical analysis of the two groups. *p* < 0.05 was considered to indicate statistical significance. *p < 0.05, **p < 0.01, ***p < 0.001, ****p < 0.0001, ns represents no significance.

## Results

### Baseline characteristics of patients

The detailed baseline characteristics of 1083 patients, including T stage, N stage, M stage, pathological stage, race, age, histological type, PAM50 molecular subtype, menopause status, and anatomic neoplasm subgroup, are listed in Table 1. Logistic analysis of the correlation between GREB1L mRNA expression and these clinical characteristics in BRCA is shown in Table 2. These results suggested that GREB1L mRNA expression may be associated with T stage, histological type, estrogen receptor (ER) status, progesterone receptor (PR) status, PAM50 subtype, and anatomic neoplasm subgroup in BRCA.

**Table 1:** Clinical characteristics of the breast cancer patients based on data from the TCGA

Characteristic	Group	Overall
*n*		1083
T stage, *n* (%)	T1	277 (25.6%)
	T2	629 (58.2%)
	T3	139 (12.9%)
	T4	35 (3.2%)
N stage, *n* (%)	N0	514 (48.3%)
	N1	358 (33.6%)
	N2	116 (10.9%)
	N3	76 (7.1%)
M stage, *n* (%)	M0	902 (97.8%)
	M1	20 (2.2%)
Pathologic stage, *n* (%)	Stage I	181 (17.1%)
	Stage II	619 (58.4%)
	Stage III	242 (22.8%)
	Stage IV	18 (1.7%)
Race, *n* (%)	Asian	60 (6%)
	Black or African American	181 (18.2%)
	White	753 (75.8%)
Age, *n* (%)	≤60	601 (55.5%)
	> 60	482 (44.5%)
Histological type, *n* (%)	Infiltrating ductal carcinoma	772 (79%)
	Infiltrating lobular carcinoma	205 (21%)
PAM50, *n* (%)	Normal	40 (3.7%)
	LumA	562 (51.9%)
	LumB	204 (18.8%)
	Her2	82 (7.6%)
	Basal	195 (18%)
Menopause status, *n* (%)	Pre	229 (23.6%)
	Peri	40 (4.1%)
	Post	703 (72.3%)
Anatomic neoplasm subgroup, *n* (%)	Left	563 (52%)
	Right	520 (48%)
Age, median (IQR)		58 (48.5, 67)

**Table 2:** Logistic analysis of the correlation between GREB1L mRNA expression and clinical characteristics in breast cancer

Characteristics	Total (N)	Odds ratio (OR)	*p* value
Age (> 60 vs. < = 60)	1083	0.997 (0.784–1.267)	0.978
Race (Black or African American &White vs. Asian)	994	1.275 (0.755–2.175)	0.367
Menopause status (Post vs. Pre & Peri)	972	0.835 (0.630–1.106)	0.208
T stage (T2 & T3 & T4 vs. T1)	1080	0.697 (0.529–0.917)	0.010
N stage (N1 & N2 & N3 vs. N0)	1064	1.225 (0.963–1.559)	0.098
M stage (M1 vs. M0)	922	0.815 (0.325–1.987)	0.652
Pathologic stage (Stage III & Stage IV vs. Stage I & Stage II)	1060	0.871 (0.658–1.153)	0.335
Histological type (Infiltrating Lobular Carcinoma vs. Infiltrating Ductal Carcinoma)	977	1.844 (1.348–2.537)	< 0.001
ER status (Positive vs. Negative & Indeterminate)	1035	11.627 (7.811–17.906)	< 0.001
PR status (Positive vs. Negative & Indeterminate)	1034	6.263 (4.669–8.482)	< 0.001
HER2 status (Positive vs. Negative & Indeterminate)	727	0.908 (0.637–1.293)	0.594
PAM50 (Basal vs. Normal & LumA & LumB & Her2)	1083	0.116 (0.074–0.176)	< 0.001
Anatomic neoplasm subgroup (Right vs. Left)	1083	0.786 (0.619–0.998)	0.048
Radiation therapy (Yes vs. No)	987	1.122 (0.872–1.443)	0.371

### The expression of GREB1L is upregulated in BRCA tissues

**Fig. 1:**
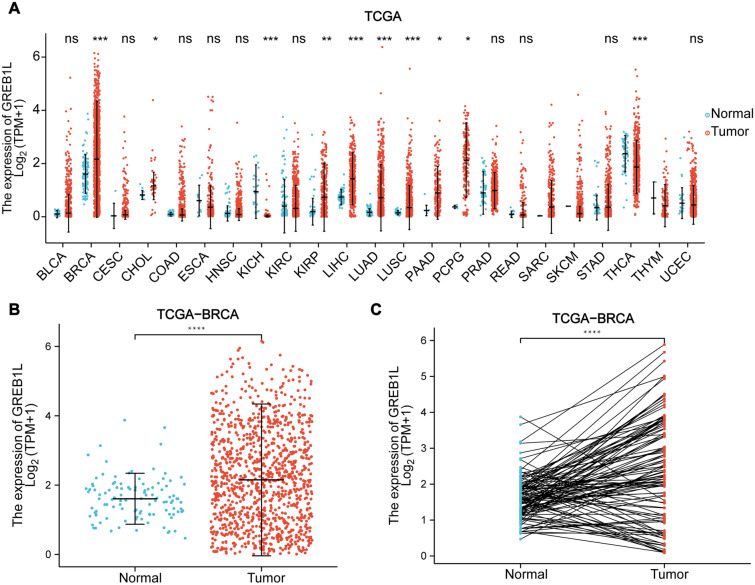
GREB1L mRNA expression in the human pancancer and breast cancer cohorts. GREB1L mRNA expression between (**A**) different human malignancies and corresponding adjacent normal tissues; **B** BRCA tissues and adjacent normal tissues (*p* = 1e-05); **C** BRCA tissues and paired adjacent normal tissues (*p* = 3.2e-08)

The differential expression analysis across cancers showed that GREB1L mRNA expression was significantly higher in BRCA (*p* = 1e-05), cholangiocarcinoma (CHOL) (*p* = 0.02), kidney renal papillary cell carcinoma (KIRP) (*p* = 7.2e-03), liver hepatocellular carcinoma (LIHC) (*p* = 2.5e-13), lung adenocarcinoma (LUAD) (*p* = 1.9e-16), lung squamous cell carcinoma (LUSC) (*p* = 1.7e-07), pancreatic adenocarcinoma (PAAD) (*p* = 0.03), and pheochromocytoma and paraganglioma (PCPG) (*p* = 0.02) tissues than in adjacent normal tissues. GREB1L expression was significantly lower in kidney chromophobe (KICH) (*p* = 8.9e-11) and thyroid carcinoma (THCA) (*p* = 2.9e-06) tissues than in normal tissues (Fig. 1A). To assess the level of GREB1L expression in BRCA patients, we analyzed the expression status data from the TCGA-BRCA dataset. The results indicated that GREB1L mRNA expression was significantly higher in BRCA tissues than in adjacent normal tissues (*p* = 1e-05) (Fig. 1B). In paired samples, the results were similar (*p* = 3.2e-08) (Fig. 1C).

### High GREB1L expression was an independent protective factor for OS in BRCA patients

**Fig. 2:**
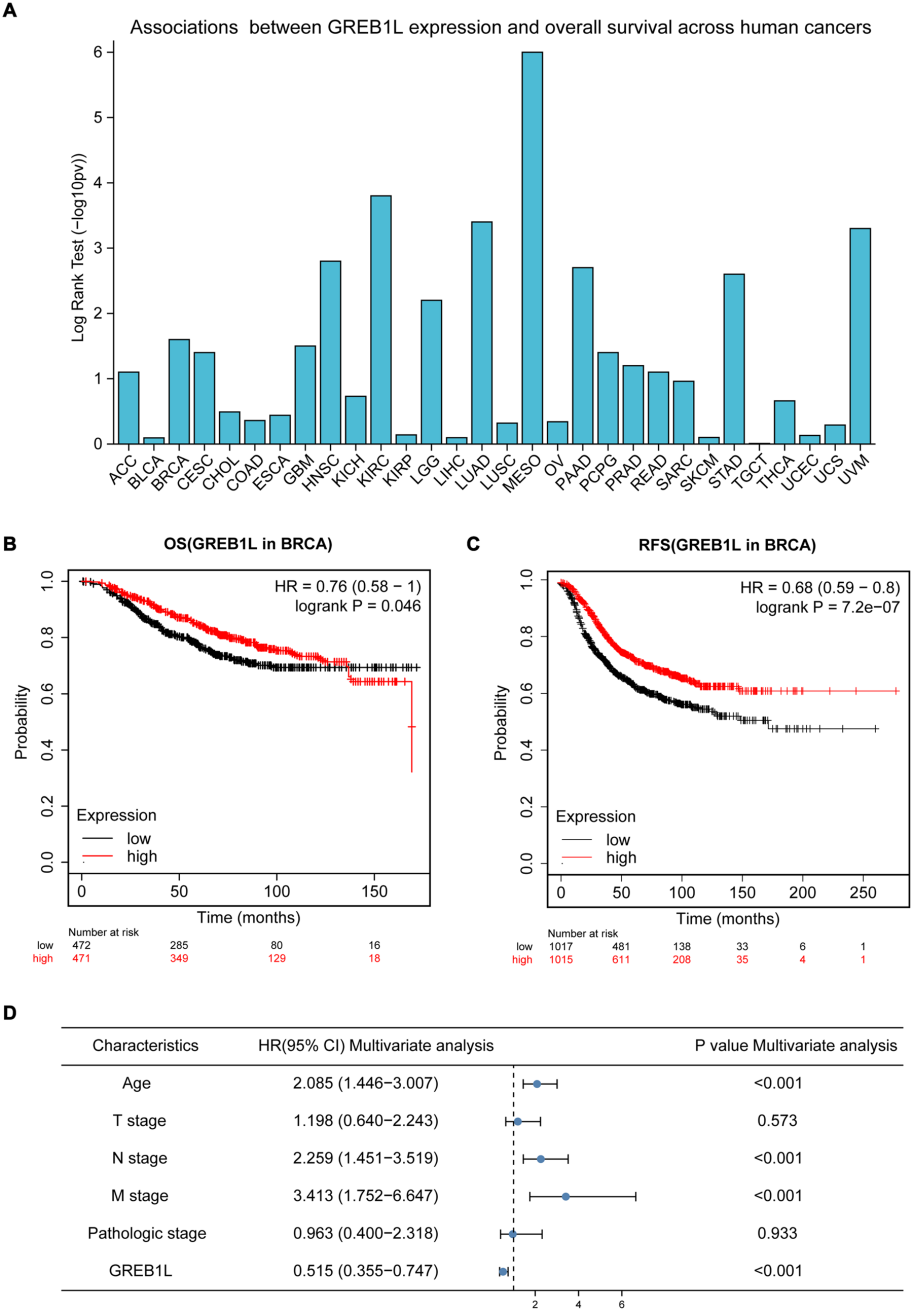
GREB1L expression is associated with a good prognosis in BRCA. **A** Bar plot for association between GREB1L expression and OS of 30 malignancies from TISIDB; **B**, **C** Kaplan‒Meier curves for OS (**B**) and RFS (**C**) in BRCA patients from Kaplan‒Meier plotter; **D** Forest plot for multivariate Cox analysis of GREB1L and clinical parameters in BRCA

TISIDB is a web portal for tumor and immune system interactions that integrates multiple heterogeneous data types [14]. Analysis on the TISIDB platform indicated that high GREB1L expression was associated with significantly longer OS in BRCA patients. BRCA ranked second across 30 human malignancies in terms of GREB1L expression level (Fig. 2A). Kaplan‒Meier plotter is a web-based survival analysis tool to assess the correlation between the expression of genes (mRNA, miRNA, protein) and patient survival for various tumor types based on databases including the Gene Expression Omnibus (GEO), European Genome-phenome Archive (EGA), and TCGA [15]. According to the Kaplan‒Meier analysis of gene chip data from the Kaplan‒Meier plotter, we found that the high GREB1L mRNA expression group for BRCA patients had a better OS (*p* = 0.046) and RFS (*p* = 7.2e-07) than the low-expression group (Fig. 2B, C). Moreover, subgroup analysis based on clinicopathological features showed that high GREB1L expression suggested better OS in patients with T3 and T4 stage disease; the N1, N2, and N3 stage disease; M0 stage disease; stage III and IV disease; age < 60 years old; infiltrating lobular carcinoma; negative HER2 status; and luminal A type disease. Interestingly, patients with high expression of GREB1L had longer OS regardless of menopausal status (Additional file 1: Figure S1). Multivariate Cox analysis indicated that older age [hazard ratio (HR) = 2.085; *p* < 0.001], high N stage (HR = 2.259; *p* < 0.001), and high M stage (HR = 3.413; *p* < 0.001) were independent risk factors for OS in BRCA patients, while a high GREB1L expression level was an independent protective factor in terms of OS (HR = 0.515; *p* < 0.001) (Fig. 2D).

### GREB1L coexpressed genes may play a role in the development of BRCA via the hedgehog signaling pathway

**Fig. 3:**
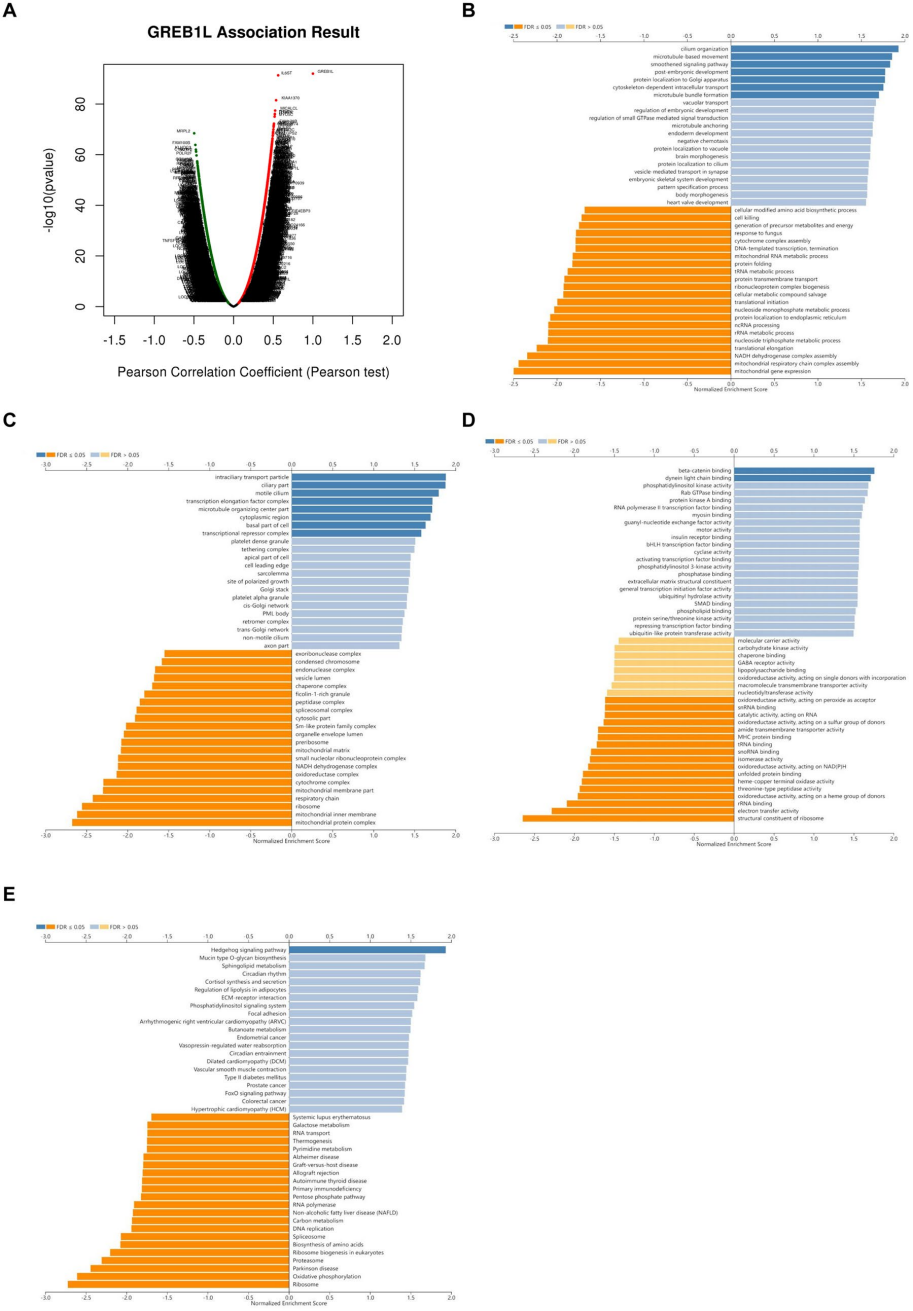
The GREB1L coexpressed genes in BRCA analyzed by the LinkedOmics database. **A** Volcano plot of the highly positively (red plots) and negatively (green plots) linked genes of GREB1L in BRCA. **B**, **E** Bar charts of the enriched terms in (**B**) GO-BP; **C** GO-CC; **D** GO-MF; and (**E**) KEGG pathway analysis by GSEA

LinkedOmics is a publicly available portal that includes multiomics data from all 32 TCGA cancer datasets and 10 Clinical Proteomics Tumor Analysis Consortium (CPTAC) cancer cohorts [16]. On the LinkedOmics platform, a total of 20,155 entries linked with GREB1L were assessed, and significantly (FDR < 0.05) positively (red dots) and negatively (green dots) correlated genes are shown in the volcano plot (Fig. 3A). The top 50 genes positively and negatively linked were selected and ranked in heatmaps (Additional file 2: Figure S2A, B). Next, we proceeded with GO enrichment analysis and KEGG pathway analysis to discover enriched functional terms and pathways. GREB1L coexpressed genes were assessed via the GSEA method. The results are shown in the bar chart. Interestingly, the significantly positively enriched terms in the GO-BP, GO-CC, and GO-MF categories were mostly related to cell movement; these included “cilium organization”, “microtubule-based movement”, “smoothened signaling pathway”, “ciliary part”, “motile cilium”, “microtubule organizing center part”, “beta-catenin binding”, and “dynein light chain binding”. However, the significantly negatively enriched terms were mostly related to energy metabolism; these included “mitochondrial respiratory chain complex assembly”, “NADH dehydrogenase complex”, “respiratory chain”, “electron transfer activity”, and “oxidoreductase activity, acting on a heme group of donors” (Fig. 3B–E). The “Hedgehog (HH) signaling pathway” was identified as the only significantly and positively enriched pathway in the KEGG pathway analysis (Fig. 3G). Previous studies have shown that the HH signaling pathway plays a crucial role in regulating embryonic development as well as the initiation and progression of multiple cancers. The HH signaling pathway exerts its biological effects by activating a G protein-coupled receptor (GPCR) family transmembrane protein called smoothened in cilium. The cilium is a kind of microtubule-based cell surface projection [17–19]. The molecular mechanisms of the HH signaling pathway covered the most significantly enriched terms revealed in the GO enrichment analysis.

### High expression of GREB1L affects the migration and invasion abilities of MCF7 and MDA-MB-231 cells

**Fig. 4:**
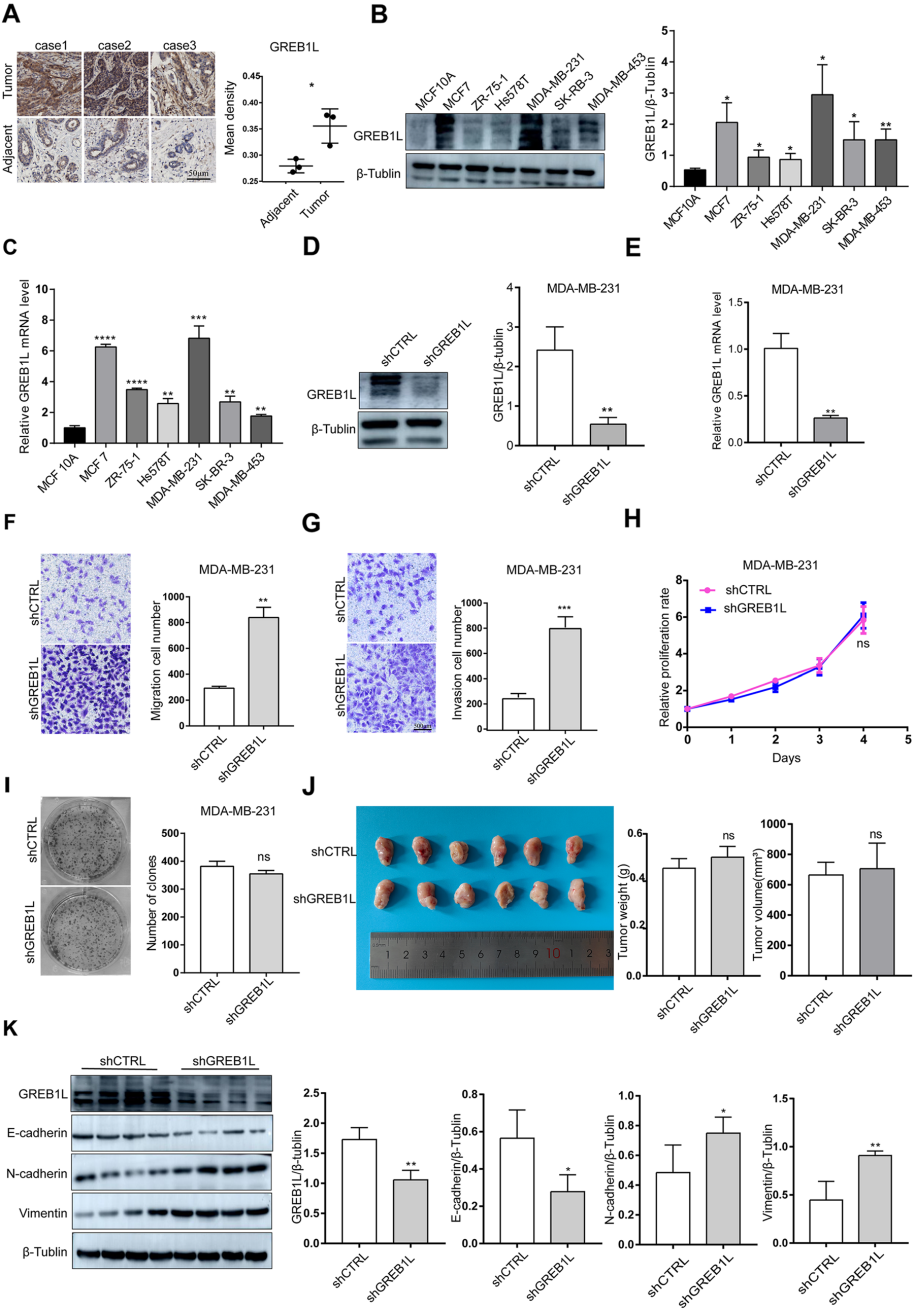
Clinical relevance of GREB1L in BRCA and its effect on MDA-MB-231 cells migration and invasion abilities. **A** Expression of GREB1L in BRCA tissues and paired adjacent tissues via immunohistochemistry (original magnification: 100 ×). (**B**) The protein level in mammary epithelial cells and BRCA cells. **C** The mRNA level in mammary epithelial cells and BRCA cells. **D**, **E** Stable knockdown of GREB1L in MDA-MB-231 cells. Western blotting (**D**) and qPCR (**E**) were used to verify the knockdown effect of GREB1L. **F**, **G** The effect of GREB1L on the migration (**F**) and invasion (**G**) abilities of MDA-MB-231 cells (original magnification: 200 ×). **H**, **I** The effect of GREB1L on the proliferation (**H**) and colony formation (**I**) abilities of MDA-MB-231 cells. **J** Images of the subcutaneous tumors formed in nude mice following injection of stable shCTRL- and shGREB1L-infected MDA-MB-231 cells. **K** The protein levels of EMT-related genes in the two groups of nude mouse tumors. *shCTRL* negative control, *shGREB1L*: shRNA for silencing GREB1L, Data are presented as the mean ± sd. of at least three independent experiments

To verify the results of our bioinformatics analysis, we randomly collected samples from three BRCA patients for immunohistochemistry analysis. We found that the expression of GREB1L in the cancer tissue was significantly higher than that in the paracancerous tissue (Fig. 4A). Moreover, we detected the protein expression levels of MCF10A normal mammary epithelial cells and MCF7, MDA-MB-231, SK-BR-3, ZR-751, and MDA-MB-453 BRCA cells. We found that the protein and mRNA expression level of GREB1L in BRCA cells were higher in BRCA cells (Fig. 4B, C). In addition, to further explore the effect of GREB1L expression level on the oncological characteristics of cells, we constructed GREB1L knockdown cell lines in MDA-MB-231 and MCF7 cells (Fig. 4D, E, Additional file 3: Figure S3A, B). Transwell migration and invasion assays detected the migration and invasion abilities of MDA-MB-231 and MCF7 cells after GREB1L knockdown. The results showed that GREB1L knockdown increased the migration and invasion abilities of BRCA cells (Fig. 4F, G**,** Additional file 3: Figure S3C, D). Next, we used CCK-8 assays to detect the effect of GREB1L knockdown on the proliferation ability of BRCA cells, and the results showed that GREB1L knockdown had no significant impact on the proliferation ability of MDA-MB-231 and MCF7 cells (Fig. 4H**,** Additional file 3: Figure S3E). Colony formation assays showed that GREB1L had no significant effect on the colony formation of MDA-MB-231 and MCF7 cells (Fig. 4I**,** Additional file 3: Figure S3F). To further evaluate whether GREB1L affects the growth of MDA-MB-231 cells in vivo, nude mice were subcutaneously transplanted with either MDA-MB-231-shCTRL or MDA-MB-231-shGREB1L cells, and we found that there was no significant difference in weight or volume between the two groups (Fig. 4J). Moreover, to verify the effect of GREB1L on the metastasis ability of breast cancer cells in vivo, we detected the protein levels of epithelial-to-mesenchymal transition (EMT) marker genes, such as E-cadherin, N-cadherin, and vimentin, in tumors isolated from mice. We found that when GREB1L was knocked down, the expression level of E-cadherin in mouse tumors decreased, and the levels of N-cadherin and vimentin showed the opposite trend, which indicated that the low expression of GREB1L promotes the occurrence of EMT (Fig. 4K). It is well known that EMT can endow cancer cells with migration and invasion characteristics, inducing stem cell-like characteristics [20]. Therefore, we believe that GREB1L can affect the metastasis of breast cancer by regulating the EMT process.

## Discussion

BRCA is the most common female malignant tumor and seriously threatens women's health. The selection of BRCA prognostic markers is of great significance for predicting the biological behavior of BRCA and guiding comprehensive treatment. In recent years, research on molecular biomarkers related to BRCA prognosis has made continuous progress, providing a basis for effectively predicting the prognosis of BRCA. For example, ER and PR have been used in clinical practice; in addition to guiding clinical classification, studies have shown that the expression of ER and PR is significantly related to the prognosis of BRCA patients. ER-positive and PR-positive patients have lower recurrence rates, higher survival rates, and better prognoses than ER-negative and PR-negative patients [21]. HER2 is an important regulator of cell growth, differentiation, and survival, and its high expression can promote the proliferation and metastasis of BRCA cells. The expression rate of HER2 was increased to 20–40% in BRCA, and this increase in expression indicated a high recurrence rate and a poor prognosis in BRCA [22, 23]. Leukemia inhibitory factor receptor (LIFR) is a BRCA metastasis suppressor that is downstream of the microRNA miR-9 and upstream of Hippo signaling. Loss of LIFR correlated with poor clinical outcomes in BRCA [24]. The expression of retinoid-inducible nuclear factor (RINF) is increased in breast tumors compared to normal tissues. Its overexpression is associated with a poor prognosis in locally advanced BRCA [25].

In this study, we discovered a novel molecule, GREB1L, which was not only associated with BRCA process but could also predict the prognosis of BRCA. Our bioinformatics analysis showed that the expression of GREB1L was higher in BRCA tissues than in adjacent normal tissues. GREB1L overexpression played a protectives role in BRCA development. Moreover, we found that, consistent with the bioinformatics results, the expression of GREB1L in BRCA tissues was higher than that in paired adjacent tissues. The protein expression level of GREB1L in BRCA cells was higher than that in mammary epithelial cells. Transwell assays showed that the knockdown of GREB1L promoted the migration and invasion of MCF7 and MDA-MB-231 cells. However, GREB1L had no significant effect on the proliferation and colony formation abilities of MCF7 and MDA-MB-231 cells. Similarly, GREB1L had no effect on the tumorigenicity of MDA-MB-231 cells in vivo, but we found by detecting the expression of EMT-related genes in nude mouse tumors that the downregulation of GREB1L promoted the EMT process of tumors, and we speculated that GREB1L can also affect the metastasis ability of BRCA cells in vivo. Thus, we believe that GREB1L is likely a protective factor for BRCA.

GREB1L is a protein-coding gene. An important paralog of this gene is growth regulating estrogen receptor binding 1 (GREB1) [26]. The function of GREB1L in BRCA has not yet been reported. Our functional enrichment analysis revealed that GREB1L is mainly associated with cell motility and energy metabolism, which suggests that GREB1L may regulate the migration ability and energy metabolism process of BRCA cells. Interestingly, the pathway enrichment analysis revealed that the HH signaling pathway was the only positively enriched pathway.

HH signaling is essential in embryonic development, tissue regeneration, and stem cell renewal [27–30]. HH pathway signaling is mediated by three ligands [sonic HH (SHH), Indian HH (IHH), and desert HH (DHH)], two receptors [patched 1 (PTCH1) and smoothened (SMO)], and three transcription factors [glioma-associated oncogene homolog (GLI)-1, GLI2, and GLI3] [31]. When there is no ligand signal, PTCH1, a transmembrane receptor on the target cell membrane, binds to SMO and inhibits SMO activity, preventing transduction. However, in the presence of an HH ligand, the HH ligand bound to PTCH1 and changed the spatial conformation of PTCH1, relieving SMO inhibition and activating the transcription factor GLI. GLI entered the nucleus, leading to the transcription of target genes and then regulating cell growth, proliferation, and differentiation [32]. Studies have shown that abnormal activation of the HH pathway is associated with the development of skin, brain, digestive tract, lung, and prostate cancer [33–37]. Abnormal reactivation of HH signaling was also reported in BRCA [38, 39]. Some scholars have found that the genes related to the HH signaling pathway play an important role in guiding the prognosis of breast cancer. This study analyzed the RFS of 3951 patients and OS of 1402 patients in the online database. They found that without considering the BRCA subgroup, high expression of SHH, HHAT, GLI1, GLI2, GLI3, and PTCH1 is associated with better RFS. High expression of HHAT is associated with better OS [40]. Our study found that GREB1L is also a gene related to the HH signaling pathway. Its high expression indicates better OS and RFS in BRCA patients, which provides a new predictive index for breast cancer prognosis.

Finally, although our study has given us an initial understanding of GREB1L in BRCA, some work remains to be completed. First, in clinical practice, we aim to determine whether the high expression of GREB1L is related to a better prognosis in BRCA patients. This aim requires long-term follow-up of our patients. Second, we need to verify whether GREB1L regulates the HH signaling pathway and regulatory mechanism in BRCA. These aims have not yet been accomplished, so we need to further explore them in the future.

## Conclusion

In conclusion, our findings suggest that GREB1L is an independent prognostic factor in BRCA. Its high expression suggests a better prognosis for patients. Inhibition of GREB1L promotes the migration and invasion abilities of MCF7 and MDA-MB-231 cells. In addition, GREB1L may play an important role in regulating the HH signaling pathway. This study demonstrated that GREB1L is a prognostic biomarker in BRCA, highlighting its potential as a predictive biomarker.

### Supplementary Information

**Additional file 1:** **Figure S1.** The prognostic value of GREB1L expression (FPKM) in BRCA based on the TCGA-BRCA dataset. **A** Kaplan‒Meier curves for OS in BRCA for all patients (n = 1082); **B** Kaplan‒Meier curves for disease-specific survival (DSS) in BRCA for all patients (n = 1062); **C** Kaplan‒Meier curves for OS in BRCA for groups with the following features: T3&T4 (n = 174); **D** N1&N2&N3 (n =706); (E) M0 (n =902); **F** pathologic stage 3 & stage 4 (n =260); **G** age<=60 (n =601); **H** histological infiltrating lobular carcinoma (n =205); **I** HER2 negative (n =558); **J** luminal A type (n =562); **K** premenopausal & perimenopausal state (n =269); **L** postmenopausal state (n =703).

**Additional file 2: Figure S2.** Top 50 genes linked with GREB1L in BRCA.** A** Positively correlated genes; **B** negatively correlated genes.

**Additional file 3: Figure S3.** The effect of GREB1L on MCF7cells migration and invasion abilities. **A**, **B** Stable knockdown of GREB1L in MCF7 cells. Western blotting (**A**) and qPCR (**B**) were used to verify the knockdown effect of GREB1L; **C**, **D** The effect of GREB1L on the migration (**C**) and invasion (**D**) abilities of MCF7 cells (original magnification: 200×); **E**, **F** The effect of GREB1L on the proliferation (**E**) and colony formation (**F**) abilities of MCF7 cells.

## Data Availability

The datasets analyzed in this study were available in the TCGA, GEO, and EGA. Additional information was available from articles or supplementary files.

## Abbreviations

BRCA: Breast cancer
HER2: Human epidermal growth factor receptor-2
PARP: Poly ADP ribose polymerase
PI3K/Akt/mTOR: Phosphoinositide 3-kinase/AKT/mammalian target of rapamycin
GREB1L: Growth regulation by estrogen in breast cancer 1 like
RAR: Retinoic acid receptor
GSEA: Gene set enrichment analysis
TCGA: The Cancer Genome Atlas
OS: Overall survival
RFS: Relapse-free survival
HR: Hazard ratio
CI: Confidence interval
GO: Gene Ontology
GO-BP: Gene Ontology biological process
GO-CC: Gene Ontology cellular component
GO-MF: Gene Ontology molecular function
KEGG: Kyoto Encyclopedia of Genes and Genomes
FDR: False discovery rate
DMEM: Dulbecco’s modified Eagle’s medium
RPMI: Roswell Park Memorial Institute
FBS: Fetal bovine serum
shRNA: Short hairpin RNA
ER: Estrogen receptor
PR: Progesterone receptor
CHOL: Cholangiocarcinoma
KIRP: Kidney renal papillary cell carcinoma
LIHC: Liver hepatocellular carcinoma
LUSC: Lung squamous cell carcinoma
PAAD: Pancreatic adenocarcinoma
PCPG: Pheochromocytoma and paraganglioma
KICH: Kidney chromophobe
THCA: Thyroid carcinoma
GEO: Gene Expression Omnibus
EGA: European Genome-phenome Archive
HR: Hazard ratio
CPTAC: Clinical Proteomics Tumor Analysis Consortium
HH: Hedgehog
GPCR: G protein-coupled receptor
LIFR: Leukemia inhibitory factor receptor
RINF: Retinoid-inducible nuclear factor
GREB1: Estrogen receptor binding 1
HH: Hedgehog
SHH: Sonic HH
IHH: Indian HH
DHH: Desert HH
PTCH1: Patched 1
SMO: Smoothened
GLI: Glioma-associated oncogene homolog
EMT: Epithelial-to-mesenchymal transition
