# Supplementary figures and images for: GREB1L overexpression is associated with good clinical outcomes in breast cancer
Source: Eur J Med Res. 2023 Nov 14;28:510. doi: 10.1186/s40001-023-01483-y (PMC10644546; doi:10.1186/s40001-023-01483-y)

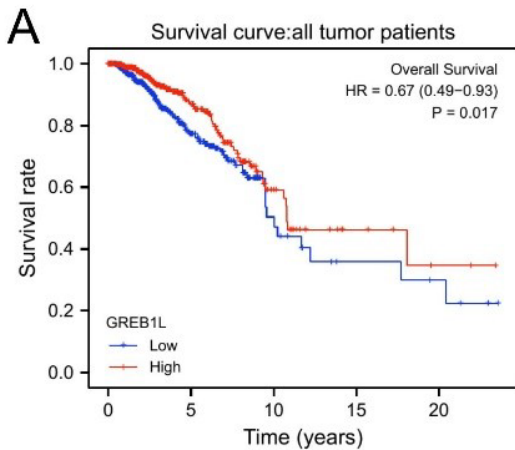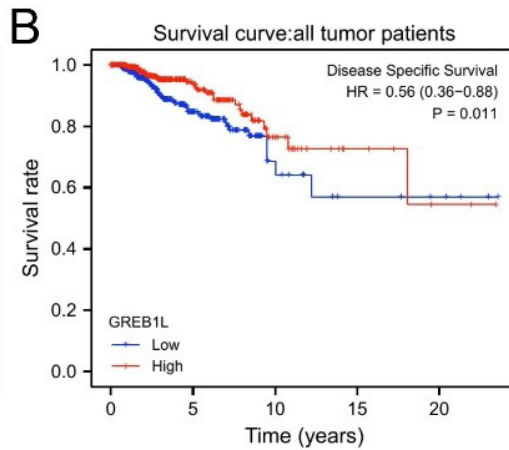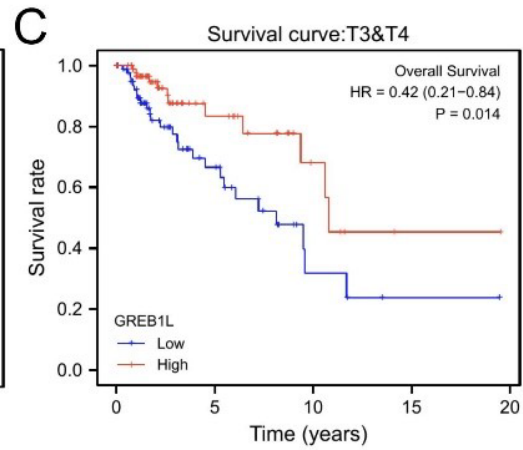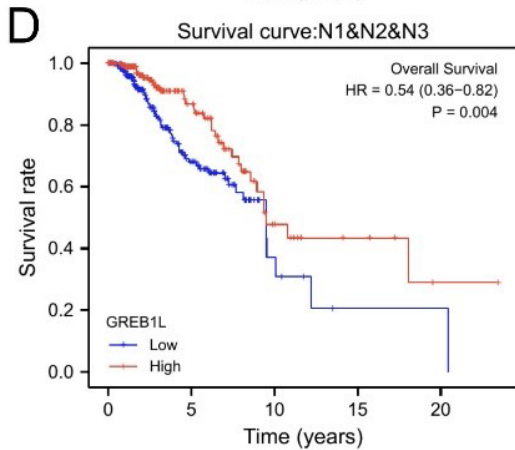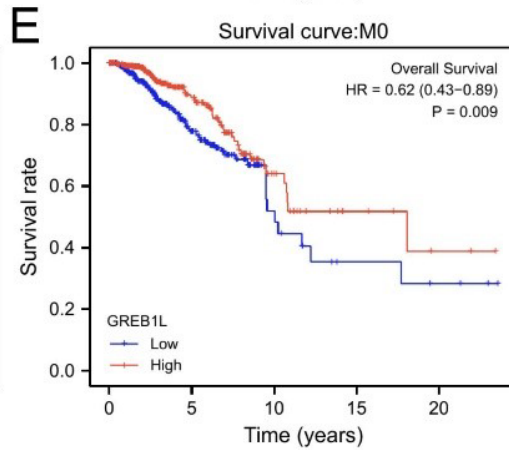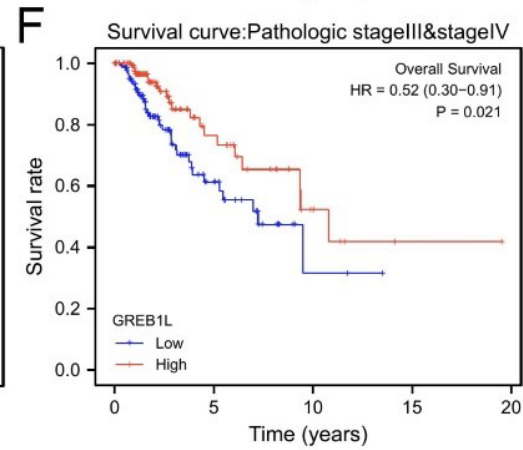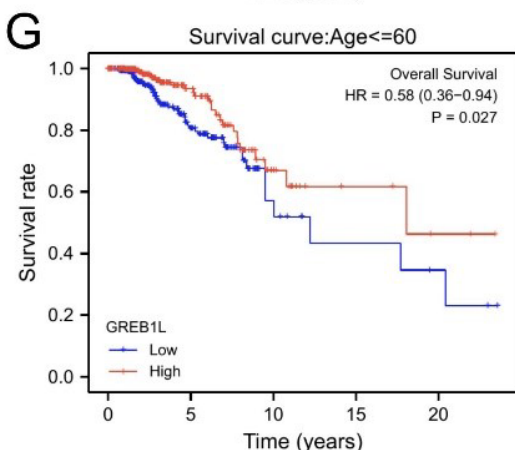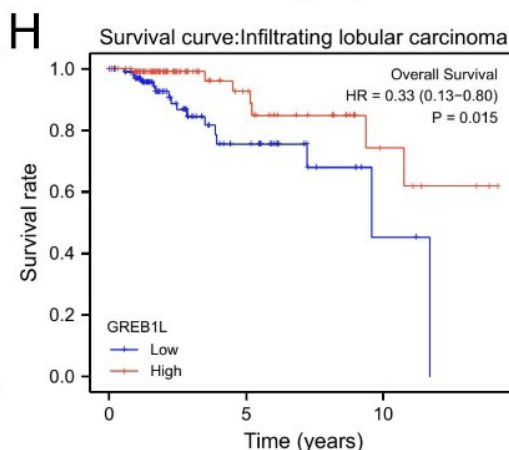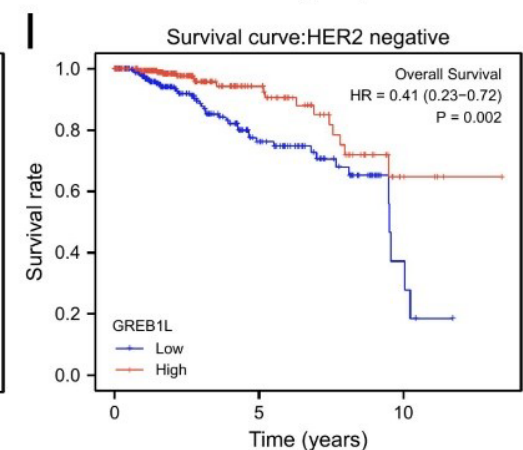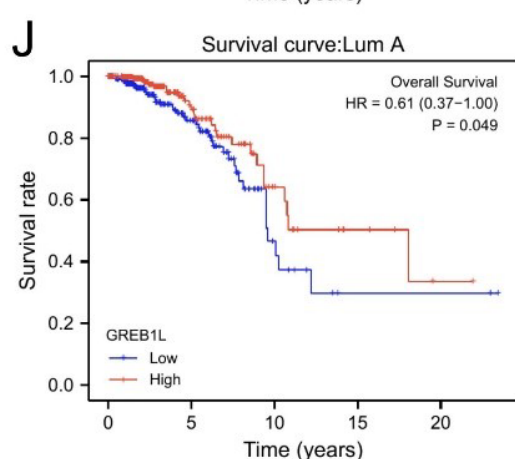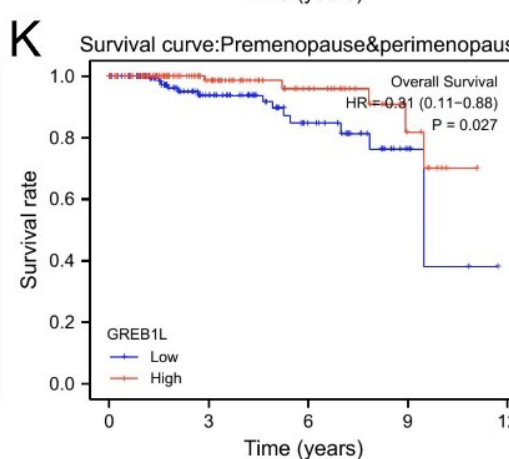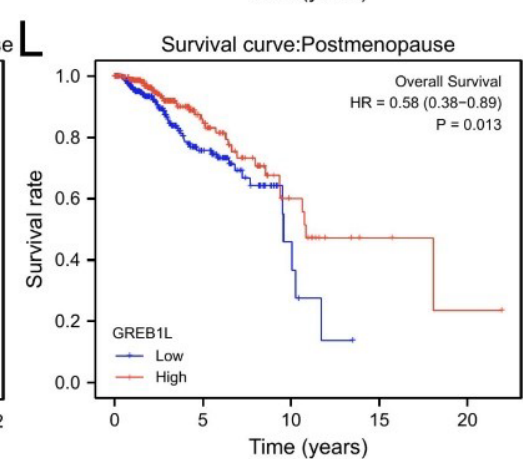

Supplement: Supplementary file 1 — Additional file 1: Figure S1. The prognostic value of GREB1L expression (FPKM) in BRCA based on the TCGA-BRCA dataset. A Kaplan‒Meier curves for OS in BRCA for all patients (n = 1082); B Kaplan‒Meier curves for disease-specific survival (DSS) in BRCA for all patients (n = 1062); C Kaplan‒Meier curves for OS in BRCA for groups with the following features: T3&T4 (n = 174); D N1&N2&N3 (n =706); (E) M0 (n =902); F pathologic stage 3 & stage 4 (n =260); G age<=60 (n =601); H histological infiltrating lobular carcinoma (n =205); I HER2 negative (n =558); J luminal A type (n =562); K premenopausal & perimenopausal state (n =269); L postmenopausal state (n =703). [file 40001_2023_1483_MOESM1_ESM.pdf]

A

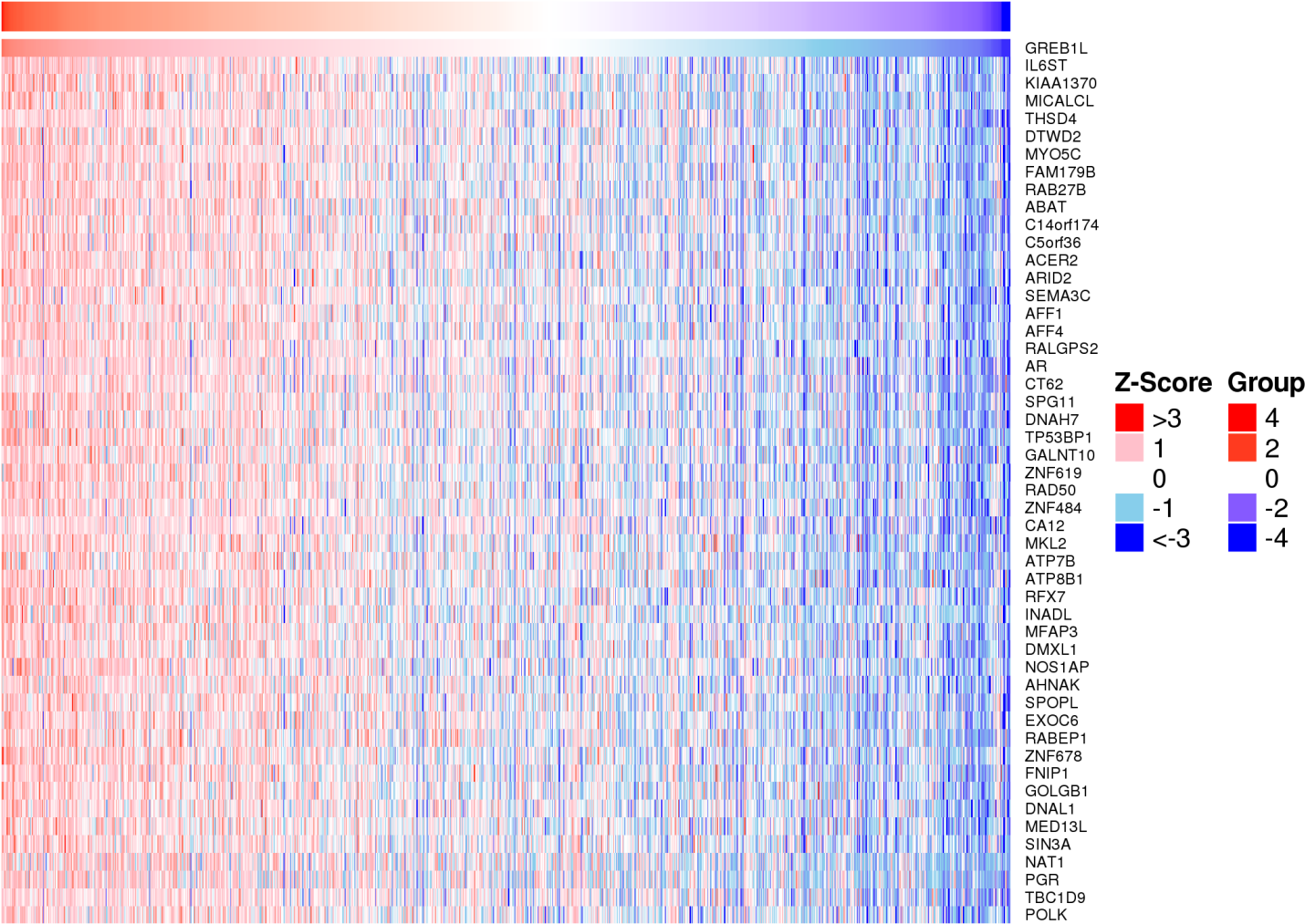

B

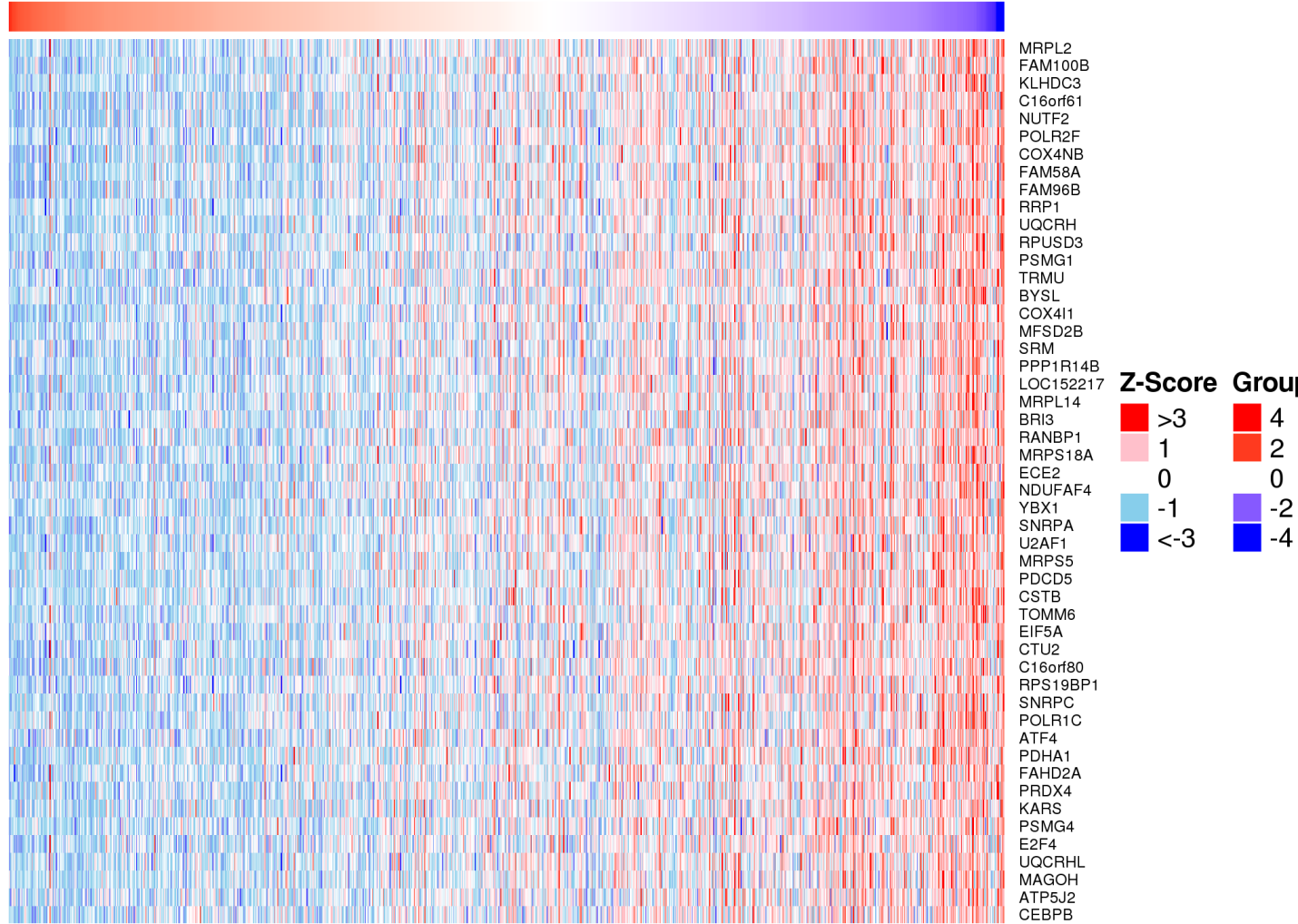

Supplement: Supplementary file 2 — Additional file 2: Figure S2. Top 50 genes linked with GREB1L in BRCA. A Positively correlated genes; B negatively correlated genes. [file 40001_2023_1483_MOESM2_ESM.pdf]

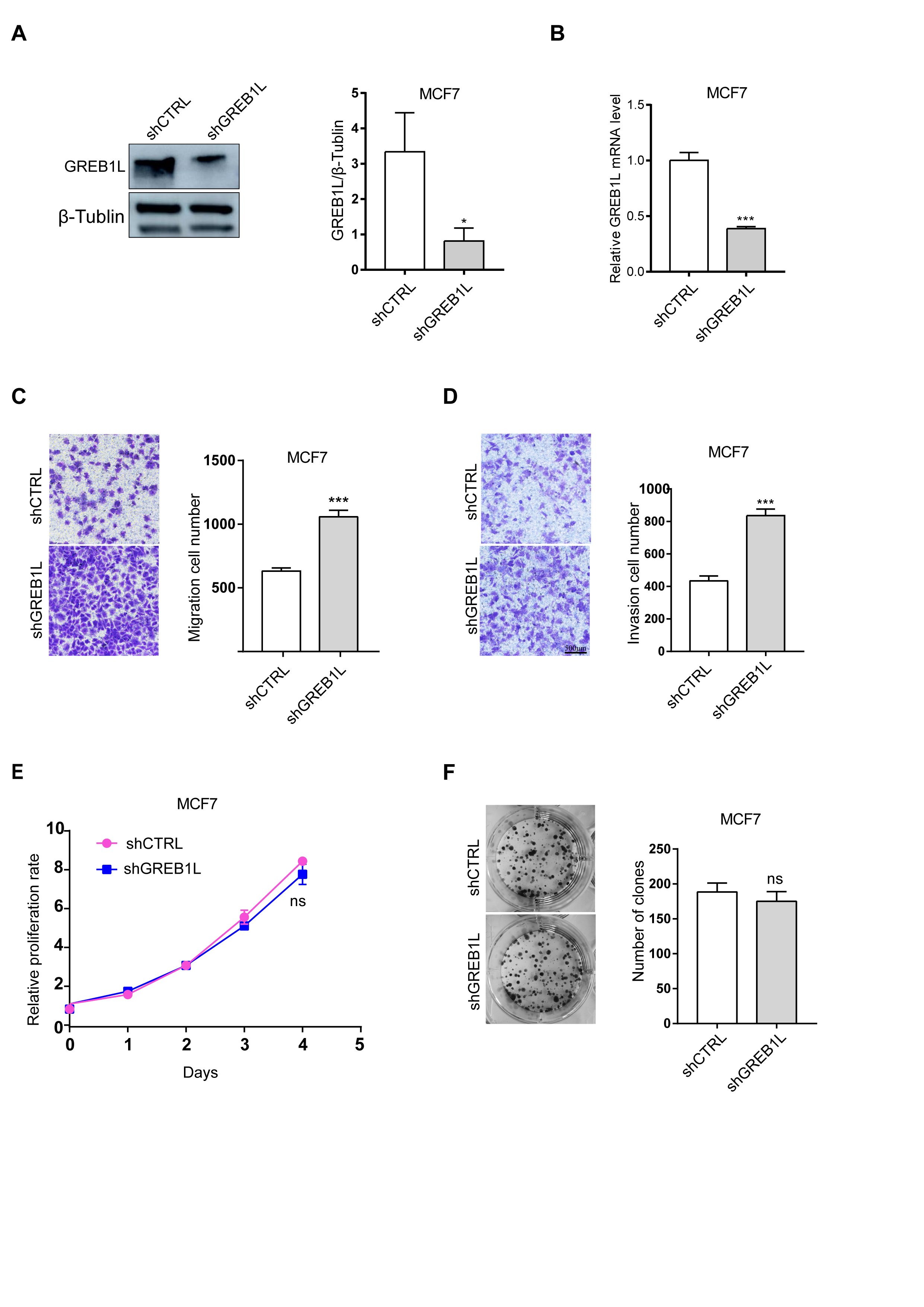

Supplement: Supplementary file 3 — Additional file 3: Figure S3. The effect of GREB1L on MCF7cells migration and invasion abilities. A, B Stable knockdown of GREB1L in MCF7 cells. Western blotting (A) and qPCR (B) were used to verify the knockdown effect of GREB1L; C, D The effect of GREB1L on the migration (C) and invasion (D) abilities of MCF7 cells (original magnification: 200×); E, F The effect of GREB1L on the proliferation (E) and colony formation (F) abilities of MCF7 cells. [file 40001_2023_1483_MOESM3_ESM.tif]
